# Genetically predicted major depression causally increases the risk of temporomandibular joint disorders

**DOI:** 10.3389/fgene.2024.1395219

**Published:** 2024-05-21

**Authors:** Shiqian Wu, Zhuo Chen, Yawen Zhao, Qiang He, Zhongxiu Yin, Hailiang Yao, Huili Liu, Lihui Yan

**Affiliations:** ^1^ Department of Stomatology, Zhengzhou Central Hospital Affiliated to Zhengzhou University, Zhengzhou University, Zhengzhou, China; ^2^ Department of Neurosurgery, West China Hospital, Sichuan University, Chengdu, China; ^3^ Queen Mary School, Nanchang University, Nanchang, China

**Keywords:** mendelian randomization, major depression, temporomandibular joint disorders, bipolar disorder, schizophrenia, causal association

## Abstract

**Objective:**

Observational studies have reported that mental disorders are comorbid with temporomandibular joint disorder (TMD). However, the causal relationship remains uncertain. To clarify the causal relationship between three common mental illnesses and TMD, we conduct this Mendelian Randomization (MR) study.

**Methods:**

The large-scale genome-wide association studies data of major depression, bipolar disorder and schizophrenia were retrieved from the Psychiatric Genomics Consortium. The summary data of TMD was obtained from the Finn-Gen consortium, including 211,023 subjects of European descent (5,668 cases and 205,355 controls). The main approach utilized was inverse variance weighting (IVW) to evaluate the causal association between the three mental disorders and TMD. Five sensitivity analyses including MR-Egger, Maximum Likelihood, Weighted median, MR. RAPS and MR-PRESSO were used as supplements. We conducted heterogeneity tests and pleiotropic tests to ensure the robustness.

**Results:**

As shown by the IVW method, genetically determined major depression was associated with a 1.65-fold risk of TMD (95% CI = 1.10–2.47, *p* < 0.05). The direction and effect size remained consistent with sensitivity analyses. The odds ratios (ORs) were 1.51 (95% CI = 0.24–9.41, *p* > 0.05) for MR-Egger, 1.60 (95% CI = 0.98–2.61, *p* > 0.05) for Weighted median, 1.68 (95% CI = 1.19–2.38, *p* < 0.05) for Maximum likelihood, 1.56 (95% CI = 1.05–2.33, *p* < 0.05) for MR. RAPS, and 1.65 (95% CI = 1.10–2.47, *p* < 0.05) for MR-PRESSO, respectively. No pleiotropy was observed (both *P* for MR-Egger intercept and Global test >0.05). In addition, the IVW method identified no significant correlation between bipolar disorder, schizophrenia and TMD.

**Conclusion:**

Genetic evidence supports a causal relationship between major depression and TMD, instead of bipolar disorder and schizophrenia. These findings emphasize the importance of assessing a patient’s depressive status in clinical settings.

## 1 Introduction

Temporomandibular disorder (TMD) is characterized by symptoms including joint sounds, dysfunctional jaw movement, and preauricular pain. This condition has become a common and frequently occurring disease in the oral and maxillofacial regions (Monteiro et al., 2011; Diniz et al., 2012). The prevalence of TMD shows a notable range of variation globally, with figures reported between 25.5% and 36.0% across different populations (Fernandes et al., 2015). Particularly in the United States, the annual economic cost associated with TMD is substantial, exceeding 100 billion dollars (Lee et al., 2021). Moreover, it significantly affects patients’ quality of life and mental health (Bair et al., 2013; Moin Anwer et al., 2021). Thus, clarifying the risk factors associated with TMD is vital, as this could potentially aid in early prevention and consequently decrease its prevalence.

Previous studies have identified several risk factors for TMD, including oral parafunctions, osteoarthritis, and other painful conditions (e.g., chronic headaches) (Ferrillo et al., 2023; Minervini et al., 2023). Furthermore, the association between TMD and mental disorders like major depression (MD), bipolar disorder (BD) and schizophrenia (SCZ) is gradually gaining attention. TMD patients were more likely to suffer from MD, with a 4.33 times greater risk than those without TMD (Liou et al., 2023). Similar findings in Korean populations reported that depression patients exhibited a 3.07-fold higher risk of developing TMD (Heo et al., 2018). Other studies suggested a potential association between BD and TMD, but lacked causal evidence (Buenaver et al., 2012; Salinas Fredricson et al., 2022). Additionally, research indicated an elevated occurrence of TMD in individuals with SCZ in contrast to the control group (83.7% vs. 67.3%) (Gurbuz et al., 2009). However, other research reported that there was no correlation between MD, BD, SCZ, and TMD (Nifosì et al., 2007; Calixtre et al., 2014; Han et al., 2018). The limitations of the aforementioned studies hinder elucidation of the causal association, including limited sample sizes and observational designs, as well as the potential for confounding bias. Furthermore, the challenge arises from the comorbidity of various mental disorders, which complicates the assessment of the individual influence of a particular mental disorder on TMD through observational designs. To achieve clarity in the causal relationship, we should conduct further research.

To address potential biases in observational designs, this study employs Mendelian Randomization (MR), often referred to as a ‘natural randomized controlled trial. MR employs genetic variants to establish causal inferences regarding the relationships between exposure factors (i.e., MD, BD, and SCZ) and outcomes (i.e., TMD) (Chen et al., 2023). The single nucleotide polymorphisms (SNPs) of the exposure factors have been randomly allocated during gestation. The association can remain unaffected by postnatal confounding factors, which could introduce bias. This helps to overcome the constraints typically associated with observational studies (Emdin et al., 2017). A clear causal is beneficial to early intervention and clinical decision-making. This approach also offers a novel perspective on TMD prevention and treatment.

## 2 Materials and methods

### 2.1 Data sources

We obtained the summary statistics of MD from seven large cohorts, including the Psychiatric Genomics Consortium (PGC), GenScot, GenScotland, GERA, iPSYCH, UKB and 23andMe. The summary-level data were from 480,359 individuals of European descent (135,458 cases and 344,901 controls) (Wray et al., 2018). Information on the diagnostic assessment of case status was available in the original reports. All individuals were assessed for a lifetime diagnosis of MD using standardized criteria. These assessments were conducted through structured interviews administered by trained interviewers, clinician-administered checklists, or reviews of medical records. The regression model incorporated sex and age, as well as the top ten principal components, as covariates to mitigate the effects of population stratification.

The summary statistics of BD were retrieved from the PGC consortium. This summary-level data was based on 51,710 participants with European ancestry (20,352 cases and 31,358 controls) (Stahl et al., 2019). Clinical information was collected via using standardized semi-structured interviews, where diagnoses of cases were made in accordance with international consensus standards. All cases reported periods of pathologically related mood elevation (mania or hypomania). Logistic regression association tests were conducted for BD within each cohort, with adjustments made for covariates, including the seven principal components, age, sex, and genetic ancestry.

The genetic association of SCZ was from PGC, representing the largest genome-wide association studies (GWAS) of schizophrenia to date. It included 127,906 European samples (52,017 cases and 75,889 controls) (Trubetskoy et al., 2022). Routinely in regression analysis, covariates such as age, gender, genotyping array, and the initial twenty principal components were taken into account.

To perform this two-sample MR analysis, we sourced TMD-related GWAS data from the FinnGen consortium (https://www.finngen.fi/en). TMD cases were diagnosed according to the revised International Classification of Diseases version 10 (K07.6), with 5,668 cases and 205,355 controls. The criteria for TMD cover a spectrum of diagnoses as follows: “snapping jaw,” “temporomandibular joint derangement,” “Costen’s syndrome or complex,” and “temporomandibular joint-pain-dysfunction syndrome.” The regression model incorporated covariates such as age, the top ten principal components, and genotyping batch. For endpoint definition details, refer to the official website (https://r9.risteys.finngen.fi/endpoints/TEMPOROMANDIB).

All MR data were publicly available (https://gwas.mrcieu.ac.uk/; accessed on 9 Oct 2023). Detailed information regarding its sample size, descent, and consortium was summarized in Table 1. Informed consent was acquired from all participants in the original genome-wide association studies. Ethical approvals and review details for GWAS datasets were available in the respective original studies.

**TABLE 1:** Characteristics of the datasets included in the analyses of psychiatric disorders and TMD.

Study characteristics	Major depression	Bipolar disorder	Schizophrenia	Temporomandibular joint disorders
PubMed ID	29700475	31043756	35396580	-
Consortium	PGC	PGC	PGC	FinnGen
Sample size	480359 (135458 cases & 344901 controls)	51710 (20352 cases & 31358 controls)	127906 (52017 cases & 75889 controls)	211023 (5668 cases & 205355 controls)
Descent	European	European	European	European
Gender	Males and females	Males and females	Males and females	Males and females
Number of SNPs	32	12	147	-
F-statistics	36.94	34.30	44.84	-
*P* for Global test	0.058	0.068	0.065	-
Intercept for Egger regression	0.0029	0.0693	0.0020	-
*P* for Egger	0.9252	0.4039	0.8618	-

### 2.2 Genetic instrument variable selection

MR has three fundamental assumptions (Luo et al., 2022; Zhu et al., 2022; Xiong et al., 2023a): (i) Relevance hypothesis: Instrumental Variables (IVs) should be closely associated with the exposure factors (i.e., MD, BD, SCZ); (ii) Independence hypothesis: IVs should be unrelated to any confounding factors; (iii) Exclusivity hypothesis (also known as the pleiotropic hypothesis): The outcome variables were exclusively influenced by IVs via the exposure factors, excluding any other pathways. We extracted SNPs from the summary data of GWAS with a significant association with MD, BD, and SCZ (meeting the relevance assumption with a threshold of *p* < 5 × 10^−8^) (Zhu et al., 2018). Then, we clumped the IVs to obtain independent SNPs (with an *r* ^*2*^ < 0.001 within a window size of 10,000 KB) (Hemani et al., 2017). A total of 16 SNPs in MD, 15 SNPs in BD, and 15 SNPs in SCZ were eliminated by excluding linkage disequilibrium, leaving 36, 16 and 158 SNPs respectively. The strength of each IV in MD, BD and SCZ was evaluated using F statistics, calculated as: F = (Beta/Se)^2^. In this formula, ‘Beta’ was the coefficient indicating the association between SNPs and traits (i.e., depression and TMD), and ‘Se’ was the standard error. An F-statistic lower than 10 for the IV indicated a potential for weak IV bias (Xiong et al., 2023b). A total of 32, 12, and 147 SNPs were used as the IVs for MD, BD and SCZ, respectively (Supplementary Tables S1–S4).

### 2.3 Statistical analyses

The effect size of each IV was estimated with using the Wald ratio and then aggregated with using the inverse variance weighting (IVW) method (Burgess et al., 2013). In cases where *p* < 0.05 in the heterogeneity test, the random-effect IVW approach was employed; otherwise, the fixed-effect IVW method was utilized (Hemani et al., 2018). In the MR-Egger method, the distance between intercept term introduced in the regression function and null could be utilized to identify directional pleiotropy. It produced effective causal effect estimation even when all IVs were invalid (Burgess and Thompson, 2017). When there was a relatively low measurement error in SNP-exposure effect, the maximum likelihood method could provide a more reliable estimate, although it might be biased due to a small sample (Yuan et al., 2024). The Weighted median method could generate more robust results when half of the IVs were invalid (Xu et al., 2024). MR robust adjustment profile score (MR. RAPS) was also employed, and the causal estimation of this model remained robust in the presence of weak IVs bias and horizontal pleiotropy (Zhao et al., 2019). MR pleiotropy residual sum and outlier (MR-PRESSO) could identify pleiotropic SNPs, remove them and then re-estimate the effect size (Verbanck et al., 2018). To evaluate the heterogeneity among IVs, we additionally performed Cochran’s Q test. Furthermore, we utilized leave-one-out analysis to demonstrate the IVs that exerted a significant impact on the estimates (Burgess et al., 2017).

This study’s analyses were conducted with R software version 4.2.2 (provided by the R Foundation for Statistical Computing, Vienna, Austria). During various phases, we utilized packages such as ‘TwoSampleMR’ and ‘MR-PRESSO’. For rectifying bias arising from multiple testing, Bonferroni correction was used in the analysis, establishing a significance threshold at *p* < 0.05/3 (two-tailed).

## 3 Results

### 3.1 Genetically predicted MD on TMD

**FIGURE 1:**
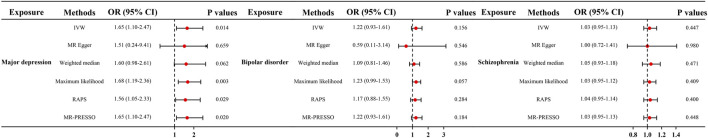
Causal effect of mental disorders on TMD in MR. OR, odds ratios; CI, confidence interval; MR, Mendelian randomization; IVW, inverse variance weighted method; RAPS, robust adjusted profile score; PRESSO, pleiotropy residual sum and outlier.

**FIGURE 2:**
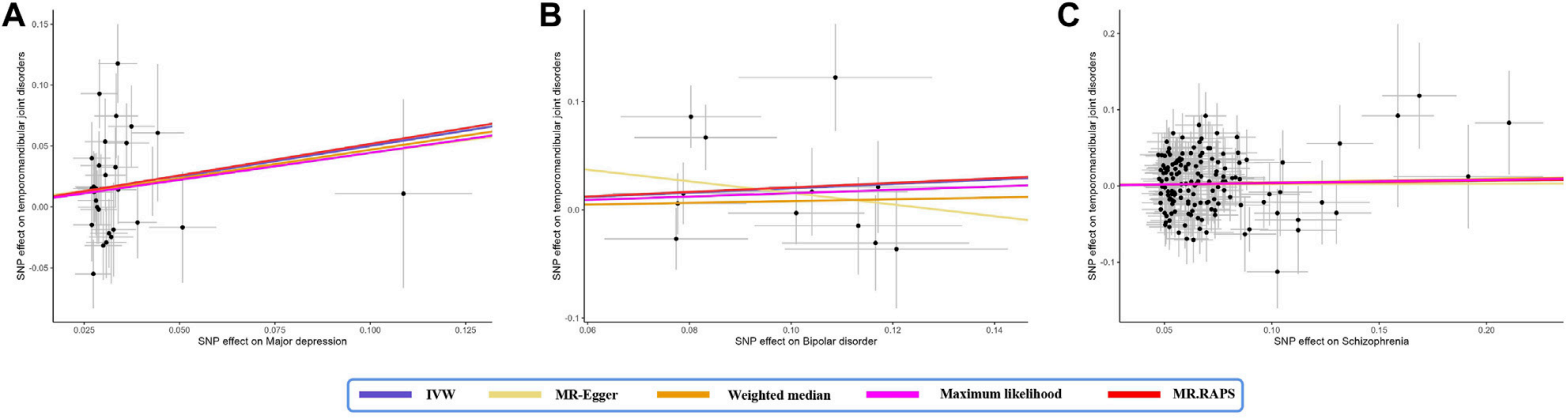
Scatter plot of the effect size of mental disorders on TMD in MR. **(A)** major depression; **(B)** bipolar disorder; **(C)** schizophrenia. SNP, single nucleotide polymorphism; IVW, inverse variance weighted method; MR, Mendelian randomization.

IVW analysis showed that genetically determined MD corresponded to a 1.65-fold risk of TMD (OR = 1.65, 95% CI = 1.10–2.47, *p* < 0.05) (Figure 1). Furthermore, the ORs obtained through the maximum likelihood method, MR. RAPS, and MR-PRESSO were significant: 1.68 (95% CI = 1.19–2.38, *p* < 0.05), 1.56 (95% CI = 1.05–2.33, *p* < 0.05), and 1.65 (95% CI = 1.10–2.47, *p* < 0.05), respectively. In contrast, the ORs obtained from MR-Egger and the weighted median method were 1.51 (95% CI = 0.24–9.41, *p* > 0.05) and 1.60 (95% CI = 0.98–2.61, *p* = 0.062), and their effect estimates were similar to the effect estimates of the above four statistical methods and consistent in direction. Figure 2A illustrates that with an increase in the SNP effect on MD, the impact of SNP on TMD also became more significant.

**FIGURE 3:**
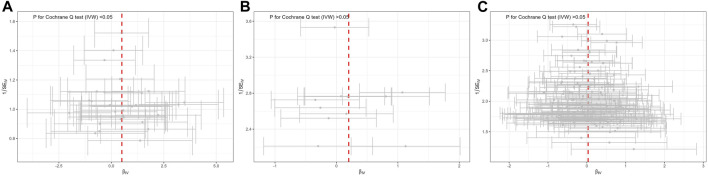
Funnel plot of potential effects of SNPs used in MR of mental disorders on TMD. **(A)** major depression; **(B)** bipolar disorder; **(C)** schizophrenia. SNP, single nucleotide polymorphism; β, the effect size; SE, the standard error of the effect size.

The Cochran’s Q tests in the MR-Egger, IVW, Maximum likelihood method revealed significant heterogeneity, with values of 45.384 (*p* = 0.036), 45.397 (*p* = 0.046), and 45.101 (*p* = 0.049), respectively (Table 2). Therefore, a random-effect IVW test was adopted for the main analytical method. The funnel plot, illustrating the heterogeneity, is presented in Figure 3A. The intercept in the MR-Egger test was 0.002 (*p* = 0.925), and *P* for Global test was 0.058, indicating the absence of directional pleiotropy (Table 1). MR-PRESSO Outlier analysis verified the absence of unknown pleiotropy as no outliers for genetic instruments were detected. Leave-one-out sensitivity analysis revealed that the causal relationship between MD and TMD remained unaffected by the exclusion of any individual SNP. This underscores the robustness of the results (Supplementary Figure S1).

**TABLE 2:** Heterogeneity test of mental disorders on TMD.

Exposures	Q values			Heterogeneity *p* values		
	MR Egger	IVW	ML	MR Egger	IVW	ML
Major depression	45.384	45.397	45.101	0.036	0.046	0.049
Bipolar disorder	17.980	19.346	19.185	0.055	0.055	0.058
Schizophrenia	172.801	172.837	172.823	0.057	0.064	0.064

MR, Mendelian randomization; IVW, inverse variance weighting; ML, maximum likelihood.

### 3.2 Genetically predicted BD on TMD

Genetically determined BD did not increase the risk of TMD (IVW: OR = 1.22, 95% CI = 0.93–1.61, *p* > 0.05) (Figure 1). The scatter plot in Figure 2B disclosed that as the impact of IVs on BD increased, there was no corresponding increase in the effect of IVs on TMD. According to the results of Cochran’s Q tests presented in Table 2 (*p* > 0.05), there were no indications of heterogeneity. The absence of heterogeneity was also depicted in the funnel plot in Figure 3B. The intercept in the MR-Egger test was 0.069 (*p* = 0.403), and the *P* for Global test was 0.068, both suggesting no directional pleiotropy, as documented in Table 1. Notably, the MR-PRESSO Outlier analysis identified no outlier SNP, affirming the absence of horizontal pleiotropy in this research.

### 3.3 Genetically predicted SCZ on TMD

As shown by the IVW approach, SCZ was not causally associated with TMD (OR = 1.03, 95% CI = 0.95–1.13, *p* > 0.05) (Figure 1). With the influence increasing of IVs impact on SCZ, there was no corresponding increase in TMD risk (Figure 2C). There was no evidence of heterogeneity, as demonstrated by the results in Table 2 (IVW: Q = 172.837, *p* = 0.064; MR-Egger: Q = 171.801, *p* = 0.057; Maximum likelihood: Q = 172.823, *p* = 0.064). Furthermore, the funnel plot displayed that genetic variations were symmetrically distributed, suggesting the absence of significant heterogeneity (Figure 3C). Furthermore, both the MR-Egger regression and the Global test revealed the absence of pleiotropy, as shown in Table 1. The MR-PRESSO Outlier did not detect any outlier SNPs, suggesting no presence of horizontal pleiotropy.

## 4 Discussion

In this research, MR methodology was applied to explore the causal association between three varying mental disorders and the risk of TMD. Our findings reveal an intriguing association: there is a significant causal link between MD and TMD as genetically determined. However, the other two mental disorders (BD and SCZ) exert no causal effect on TMD. Therefore, it is imperative to evaluate the mental wellbeing of TMD patients in clinical practice, as this can contribute to enhancing their treatment and long-term prognosis.

This research sheds light on the connection between MD and TMD. Early observations help elucidate the link between MD and TMD (Giannakopoulos et al., 2010; De La Torre Canales et al., 2018; Sójka et al., 2019). An epidemiological study demonstrated that the prevalence of MD rises in tandem with the incidence of TMD pain-related injuries. The prevalence varied, showing a range from 16.7% in TMD individuals with no disability to 53.8% in those exhibiting high disability and severe restrictive injuries (Maísa Soares and Rizzatti-Barbosa, 2015). In a prospective study with 3,006 individuals, Kindler et al. (2012) confirmed that patients with MD exhibited a 1.95-fold higher risk of TMD than controls. Corresponding results were similarly observed in the studies conducted by Simoen et al. (2020). However, some studies contradicted this association (Pfau et al., 2009; Pizolato et al., 2013), highlighting the potential for discrepancies in findings due to differences in methodologies. To enhance data reliability and account for potential data category overlaps, we selected two distinct datasets (PGC and FinnGen). Our findings contribute to a broader comprehension of the effects of MD on TMD and offer additional causal evidence to inform clinical interventions.

However, the specific mechanism between depression and TMD still remains unclear. Differences in the regulation of the hypothalamus-pituitary-adrenal (HPA) axis in response to depressive symptoms could result in varying alterations in the body’s stress and alarm systems (Heim et al., 2008). These chronic regulatory processes can lead to ongoing muscle hyperactivity, progressively damaging the joint and ultimately manifesting as symptoms of TMD (Staniszewski et al., 2018). Depression may influence different gene polymorphisms, including COMT, ADRB2, and HTR1A genes, which are associated with chronic joint pain and TMD (Schwahn et al., 2012; Slade et al., 2013; Bonato et al., 2021). TNF-α, potentially serving as an inflammatory cytokine, mediates the relationship between MD and TMD. Elevated serum levels of TNF-α in individuals with MD could contribute, either directly or indirectly, to the development of TMD. Moreover, the heightened excitability of the HPA axis may, to some extent, affect the activity of the TNF-α system (Emshoff et al., 2000; Shrivastava et al., 2021). Additionally, if certain risk factors, such as genetic polymorphisms, exert differential impacts on muscle and joint discomfort, the detected specificity could stem from the distinct effect modification of depressive symptoms. Therefore, a more in-depth study is needed to unravel the complex mechanism between MD and TMD.

Our MR results indicate a lack of significant genetic influence on TMD risk in relation to BD or SCZ. In a prospective case-control study involving 366,437 Swedish individuals, Salinas Fredricson et al. (2022) reported a 1.40-fold higher risk of BD in patients with TMD than controls. However, there was no significant association with SCZ found in their study. Other observational research has indicated a potential correlation between SCZ and a heightened risk for TMD (Velasco-Ortega et al., 2005; de Araújo et al., 2014). Currently, our research does not confirm a direct causal relationship among BD, SCZ, and TMD. Nevertheless, the OR for BD is 1.22, which is greater than 1, and this result can still undergo further validation in a larger sample size.

There are several advantages to our research. Firstly, conducting randomized controlled trials to study disease development would be ethically problematic, as it would delay treatment for potentially ill patients. In contrast, MR analysis provides a more ethical approach, allowing us to utilize public data readily and control postnatal confounding factors in traditional observational studies. Secondly, our research, grounded in a European demographic, which helps reduce the potential of stratification bias. Thirdly, we utilize a large sample of GWAS datasets to enhance statistical power for both exposure and outcome. This includes the MD GWAS dataset (n = 480,359) and the TMD GWAS summary dataset (n = 211,023). Moreover, employing six different MR analysis methods, no substantial deviations across various methods were revealed, thus reinforcing the robustness of our results.

Nevertheless, some limitations need to be acknowledged. The further validation in diverse populations is warranted and not limited to European populations. Then, the causal relationship between mental illness and TMD can be influenced by various confounding factors. There is a complex genetic interplay between mental illness and other traits such as sleep quality, autoimmune diseases (e.g., rheumatoid arthritis), physical factors, and pain conditions (e.g., chronic headaches) (Sanders et al., 2013). Physical factors are genetically related to depression, and there is substantial genetic overlap with autoimmune diseases. These factors could potentially confound our research (Euteneuer et al., 2012; Fillingim et al., 2013; Ishigaki et al., 2022). In addition, the diagnostic criteria for TMD, as established by the DC/TMD, classify TMD into two main categories: painful disorders and joint disorders, with nine common diagnoses (Schiffman and Ohrbach, 2016; Khan et al., 2023). In future investigations these distinctions should be taken into account.

While the MR method offers advantages in estimating causality, multicenter randomized controlled trials with a large sample size continue to be the gold standard for confirming causality. Therefore, interpreting the results from MR studies should be objectivity.

## 5 Conclusion

In summary, we provide genetic evidence supporting a causal association between MD and TMD. Nonetheless, the underlying mechanism connecting MD and TMD warrants further investigation. Our study does not identify causal relationships between BD, SCZ, and TMD within the European population. Intervening MD at an early stage could potentially reduce the incidence of TMD. As a result, proactive interventions should be considered to mitigate the risk of TMD.

## Data Availability

The datasets presented in this study can be found in online repositories. The names of the repository/repositories and accession number(s) can be found in the article/Supplementary Material.
