# Supplementary material for: Genetically predicted major depression causally increases the risk of temporomandibular joint disorders
Source: Front Genet. 2024 May 21;15:1395219. doi: 10.3389/fgene.2024.1395219 (PMC11148344; doi:10.3389/fgene.2024.1395219)
Supplement: Supplementary file 1 [file Table1.docx]

**Supplementary table 1 Details of the GWASs**

| **GWAS ID** | **Trait** | **Consortium** | **Population** | **Participants** | **PMID** |
| --- | --- | --- | --- | --- | --- |
| **ieu-a-1187** | Major depression | PGC | European | 480,359 | 29700475 |
| **ieu-b-41** | Bipolar Disorder | PGC | European | 51,710 | 31043756 |
| **ieu-b-5102** | Schizophrenia | PGC | European | 127,906 | 35396580 |
