# Supplementary material for: Genetically predicted major depression causally increases the risk of temporomandibular joint disorders
Source: Front Genet. 2024 May 21;15:1395219. doi: 10.3389/fgene.2024.1395219 (PMC11148344; doi:10.3389/fgene.2024.1395219)
Supplement: Supplementary file 2 [file Table2.docx]

**Supplementary table 2 instrumental variables for major depression**

|  | SNP | EA | OA | Beta | Se | P value |
| --- | --- | --- | --- | --- | --- | --- |
| 1 | rs10149470 | G | A | 0.0289964 | 0.0049 | 3.05E-09 |
| 2 | rs10950398 | A | G | 0.0274984 | 0.0049 | 2.55E-08 |
| 3 | rs10959913 | G | T | -0.0333961 | 0.0057 | 5.06E-09 |
| 4 | rs11135349 | C | A | 0.0293979 | 0.0048 | 1.09E-09 |
| 5 | rs11643192 | A | C | 0.0270021 | 0.0049 | 3.36E-08 |
| 6 | rs11663393 | A | G | 0.0278 | 0.0049 | 1.65E-08 |
| 7 | rs11682175 | C | T | 0.0281012 | 0.0048 | 4.68E-09 |
| 8 | rs1226412 | T | C | 0.0332026 | 0.0059 | 2.38E-08 |
| 9 | rs12552 | G | A | -0.0428966 | 0.0048 | 6.07E-19 |
| 10 | rs12666117 | A | G | 0.0274011 | 0.0048 | 1.35E-08 |
| 11 | rs12958048 | G | A | -0.0338022 | 0.0051 | 3.61E-11 |
| 12 | rs1354115 | A | C | 0.0275957 | 0.0049 | 2.37E-08 |
| 13 | rs1363104 | G | C | -0.0314018 | 0.0048 | 7.38E-11 |
| 14 | rs1432639 | A | C | 0.0389995 | 0.005 | 4.55E-15 |
| 15 | rs159963 | A | C | -0.0270013 | 0.0049 | 3.19E-08 |
| 16 | rs17727765 | C | T | 0.0507987 | 0.0088 | 8.51E-09 |
| 17 | rs1806153 | T | G | 0.0361005 | 0.0059 | 1.18E-09 |
| 18 | rs2005864 | T | C | 0.0281987 | 0.0049 | 6.73E-09 |
| 19 | rs2389016 | T | C | 0.0305001 | 0.0053 | 1.02E-08 |
| 20 | rs247910 | G | A | 0.031501 | 0.0049 | 1.07E-10 |
| 21 | rs34215985 | G | C | 0.037297 | 0.0063 | 3.13E-09 |
| 22 | rs4074723 | C | A | 0.0270013 | 0.0049 | 3.12E-08 |
| 23 | rs4904738 | C | T | 0.0289037 | 0.0049 | 2.57E-09 |
| 24 | rs61867293 | T | C | -0.0374008 | 0.0061 | 6.97E-10 |
| 25 | rs62099069 | T | A | 0.0278955 | 0.0049 | 1.31E-08 |
| 26 | rs6905391 | A | G | -0.0442968 | 0.0069 | 1.35E-10 |
| 27 | rs7198928 | C | T | -0.0284028 | 0.005 | 1.00E-08 |
| 28 | rs7430565 | A | G | -0.0288008 | 0.0048 | 2.87E-09 |
| 29 | rs76485002 | G | A | -0.108702 | 0.018 | 1.60E-09 |
| 30 | rs7856424 | T | C | -0.0306035 | 0.0053 | 8.48E-09 |
| 31 | rs8025231 | C | A | 0.0338981 | 0.0048 | 2.36E-12 |
| 32 | rs8063603 | A | G | -0.0307995 | 0.0053 | 6.86E-09 |
| 33 | rs915057 | G | A | 0.0299954 | 0.0049 | 7.61E-10 |
| 34 | rs9402472 | A | G | 0.0326995 | 0.0059 | 2.78E-08 |
| 35 | rs9402472 | A | G | 0.0326995 | 0.0059 | 2.78E-08 |
| 36 | rs9427672 | G | A | 0.0320997 | 0.0058 | 3.12E-08 |
