# Supplementary figures and images for: Genetically predicted major depression causally increases the risk of temporomandibular joint disorders
Source: Front Genet. 2024 May 21;15:1395219. doi: 10.3389/fgene.2024.1395219 (PMC11148344; doi:10.3389/fgene.2024.1395219)

**Supplementary figure 1: leave-one-out sensitivity analysis for MD on TMD**


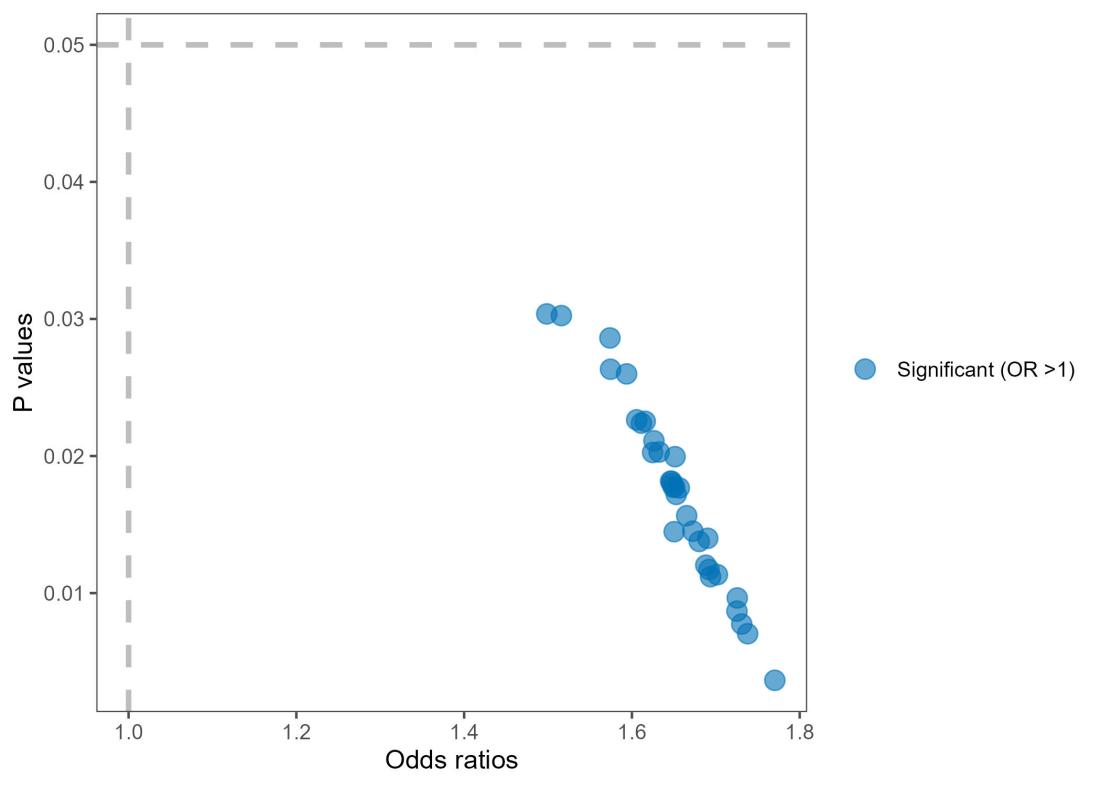

Supplement: Supplementary file 3 [file Table5.docx]
