# Supplementary material for: Genetically predicted major depression causally increases the risk of temporomandibular joint disorders
Source: Front Genet. 2024 May 21;15:1395219. doi: 10.3389/fgene.2024.1395219 (PMC11148344; doi:10.3389/fgene.2024.1395219)
Supplement: Supplementary file 4 [file Table3.docx]

|  | SNP | EA | OA | Beta | Se | P value |
| --- | --- | --- | --- | --- | --- | --- |
| 1 | rs10744560 | T | C | 0.083201 | 0.014 | 2.92E-09 |
| 2 | rs111444407 | T | C | 0.1166 | 0.0184 | 2.40E-10 |
| 3 | rs11724116 | T | C | -0.104095 | 0.0188 | 3.27E-08 |
| 4 | rs13231398 | C | G | -0.1207 | 0.0219 | 3.36E-08 |
| 5 | rs17150022 | C | T | 0.113202 | 0.0204 | 2.70E-08 |
| 6 | rs174592 | G | A | 0.0774 | 0.0141 | 3.66E-08 |
| 7 | rs2071044 | T | C | -0.077702 | 0.0135 | 9.09E-09 |
| 8 | rs2314398 | G | C | -0.084102 | 0.0144 | 5.92E-09 |
| 9 | rs329319 | G | A | -0.078802 | 0.0139 | 1.54E-08 |
| 10 | rs55648125 | G | A | 0.117096 | 0.0215 | 4.92E-08 |
| 11 | rs5758065 | G | C | -0.074402 | 0.0135 | 3.23E-08 |
| 12 | rs73496688 | A | T | 0.108702 | 0.019 | 1.05E-08 |
| 13 | rs884301 | T | C | 0.080298 | 0.0138 | 5.80E-09 |
| 14 | rs9834970 | C | T | 0.101003 | 0.0134 | 5.53E-14 |

**Supplementary table 3 instrumental variables for bipolar disorder**
