# Supplementary material for: Genetically predicted major depression causally increases the risk of temporomandibular joint disorders
Source: Front Genet. 2024 May 21;15:1395219. doi: 10.3389/fgene.2024.1395219 (PMC11148344; doi:10.3389/fgene.2024.1395219)
Supplement: Supplementary file 5 [file Table4.docx]

**Supplementary table 4: instrumental variables for schizophrenia**

|  | SNP | EA | OA | Beta | Se | P value |
| --- | --- | --- | --- | --- | --- | --- |
| 1 | rs1000237 | A | T | 0.0732053 | 0.0089 | 2.80E-16 |
| 2 | rs10035564 | G | A | 0.0668024 | 0.0092 | 4.38E-13 |
| 3 | rs10086619 | G | A | 0.0722052 | 0.0116 | 4.97E-10 |
| 4 | rs10103330 | A | T | 0.067305 | 0.0108 | 5.07E-10 |
| 5 | rs10103330 | A | T | 0.067305 | 0.0108 | 5.07E-10 |
| 6 | rs10117 | A | G | -0.0549994 | 0.0088 | 4.66E-10 |
| 7 | rs10861176 | A | G | 0.0555021 | 0.0098 | 1.59E-08 |
| 8 | rs10876446 | C | G | 0.0540022 | 0.0094 | 1.03E-08 |
| 9 | rs10957321 | A | G | 0.0475949 | 0.0086 | 3.48E-08 |
| 10 | rs11027839 | C | A | 0.0515038 | 0.0086 | 2.40E-09 |
| 11 | rs11136325 | A | G | -0.0537967 | 0.0091 | 3.05E-09 |
| 12 | rs11165867 | T | C | 0.0743034 | 0.0116 | 1.30E-10 |
| 13 | rs11191580 | C | T | -0.131703 | 0.0155 | 1.77E-17 |
| 14 | rs11210892 | A | G | -0.0635005 | 0.0091 | 2.68E-12 |
| 15 | rs113264400 | C | T | 0.112296 | 0.0202 | 2.86E-08 |
| 16 | rs11587347 | G | C | 0.103895 | 0.0147 | 1.53E-12 |
| 17 | rs11664298 | A | G | 0.0773995 | 0.0108 | 8.94E-13 |
| 18 | rs11693094 | T | C | -0.054403 | 0.0087 | 4.29E-10 |
| 19 | rs11696755 | C | T | 0.0636962 | 0.011 | 7.26E-09 |
| 20 | rs11740474 | T | A | 0.0536962 | 0.0088 | 1.13E-09 |
| 21 | rs11941714 | A | G | -0.0515957 | 0.0093 | 3.07E-08 |
| 22 | rs1198588 | T | A | 0.102598 | 0.0108 | 1.73E-21 |
| 23 | rs12129573 | A | C | 0.0777994 | 0.0089 | 2.28E-18 |
| 24 | rs12138231 | A | T | 0.0669949 | 0.0116 | 7.99E-09 |
| 25 | rs12151767 | A | G | -0.0611045 | 0.0086 | 1.31E-12 |
| 26 | rs12285419 | A | C | 0.0849045 | 0.011 | 1.05E-14 |
| 27 | rs12293670 | G | A | -0.0704957 | 0.0092 | 1.56E-14 |
| 28 | rs12303743 | C | G | 0.0874988 | 0.0145 | 1.59E-09 |
| 29 | rs12489270 | C | T | 0.0579046 | 0.0089 | 7.47E-11 |
| 30 | rs12652777 | C | T | -0.0487997 | 0.0086 | 1.52E-08 |
| 31 | rs12712510 | C | T | -0.0574006 | 0.0087 | 5.14E-11 |
| 32 | rs12771371 | A | G | -0.0524027 | 0.0093 | 1.94E-08 |
| 33 | rs12833624 | T | C | 0.0501992 | 0.009 | 2.77E-08 |
| 34 | rs12877581 | C | G | 0.0596014 | 0.0099 | 1.80E-09 |
| 35 | rs12883788 | T | C | 0.0613011 | 0.0087 | 1.86E-12 |
| 36 | rs13011472 | G | C | 0.070401 | 0.0087 | 4.28E-16 |
| 37 | rs13016542 | C | T | -0.0883039 | 0.0129 | 8.28E-12 |
| 38 | rs13107325 | T | C | 0.158703 | 0.0168 | 2.90E-21 |
| 39 | rs13195636 | C | A | -0.210504 | 0.0159 | 6.55E-40 |
| 40 | rs13233308 | T | C | -0.0487044 | 0.0086 | 1.75E-08 |
| 41 | rs132582 | T | C | -0.0509972 | 0.0086 | 3.26E-09 |
| 42 | rs1427633 | C | G | -0.0483043 | 0.0088 | 4.10E-08 |
| 43 | rs1430894 | T | C | 0.0532953 | 0.0086 | 6.15E-10 |
| 44 | rs145071536 | C | T | 0.0851005 | 0.012 | 1.62E-12 |
| 45 | rs1451488 | G | A | 0.0708947 | 0.0087 | 4.47E-16 |
| 46 | rs149165 | G | T | -0.0481995 | 0.0087 | 3.00E-08 |
| 47 | rs1540840 | C | G | -0.0556996 | 0.0093 | 2.21E-09 |
| 48 | rs1593304 | G | A | 0.0641013 | 0.0111 | 7.45E-09 |
| 49 | rs1604060 | G | A | 0.0772051 | 0.014 | 3.24E-08 |
| 50 | rs1611236 | A | G | -0.0551035 | 0.0096 | 8.47E-09 |
| 51 | rs1615350 | T | C | -0.0736036 | 0.0098 | 4.92E-14 |
| 52 | rs167924 | G | A | 0.0501992 | 0.009 | 2.34E-08 |
| 53 | rs16851048 | C | T | 0.0744973 | 0.0107 | 4.15E-12 |
| 54 | rs16867571 | G | A | -0.0657035 | 0.0104 | 2.68E-10 |
| 55 | rs17194490 | T | G | 0.0781994 | 0.0116 | 1.80E-11 |
| 56 | rs17731 | A | G | 0.0523992 | 0.0089 | 4.37E-09 |
| 57 | rs1860002 | T | C | -0.0837987 | 0.0087 | 1.04E-21 |
| 58 | rs187557 | T | C | -0.0666956 | 0.0119 | 2.03E-08 |
| 59 | rs1881046 | T | G | -0.0507026 | 0.0092 | 3.39E-08 |
| 60 | rs1892346 | A | T | 0.0484027 | 0.0088 | 3.56E-08 |
| 61 | rs1901512 | C | T | -0.058401 | 0.0094 | 5.72E-10 |
| 62 | rs1914399 | G | C | -0.0491044 | 0.0087 | 1.40E-08 |
| 63 | rs1915019 | G | A | -0.0570984 | 0.0098 | 6.57E-09 |
| 64 | rs1953205 | A | T | 0.0499048 | 0.0089 | 2.21E-08 |
| 65 | rs2053079 | G | A | 0.0598986 | 0.0101 | 3.01E-09 |
| 66 | rs2078266 | G | A | -0.0696007 | 0.0126 | 2.94E-08 |
| 67 | rs215412 | A | G | 0.0577033 | 0.0091 | 2.69E-10 |
| 68 | rs2167378 | T | C | -0.0648978 | 0.0087 | 7.30E-14 |
| 69 | rs217336 | A | C | -0.0503033 | 0.0087 | 8.05E-09 |
| 70 | rs2332700 | G | C | -0.0750982 | 0.0099 | 3.88E-14 |
| 71 | rs2333321 | G | A | -0.0712038 | 0.0105 | 1.25E-11 |
| 72 | rs2381411 | C | T | 0.050399 | 0.0088 | 1.25E-08 |
| 73 | rs2455415 | T | C | 0.0494949 | 0.0088 | 1.69E-08 |
| 74 | rs2456020 | T | C | -0.0815984 | 0.0102 | 1.13E-15 |
| 75 | rs2514218 | T | C | -0.0704957 | 0.0092 | 1.35E-14 |
| 76 | rs2696466 | G | A | -0.0611986 | 0.0092 | 2.64E-11 |
| 77 | rs2710323 | C | T | -0.0784044 | 0.0086 | 1.23E-19 |
| 78 | rs2815731 | A | C | -0.0600033 | 0.0091 | 4.39E-11 |
| 79 | rs2909457 | A | G | -0.0489997 | 0.0087 | 1.48E-08 |
| 80 | rs2999392 | T | C | 0.0517987 | 0.0094 | 3.05E-08 |
| 81 | rs308697 | A | C | -0.0501036 | 0.0087 | 8.83E-09 |
| 82 | rs34555420 | T | G | -0.168696 | 0.0173 | 1.54E-22 |
| 83 | rs34555420 | T | G | -0.168696 | 0.0173 | 1.54E-22 |
| 84 | rs35351411 | C | A | 0.0635044 | 0.0087 | 2.21E-13 |
| 85 | rs35426637 | T | G | -0.0622985 | 0.0093 | 2.15E-11 |
| 86 | rs35734242 | C | T | 0.050704 | 0.0089 | 1.37E-08 |
| 87 | rs3739118 | A | G | -0.057004 | 0.0095 | 2.36E-09 |
| 88 | rs3770754 | G | C | -0.052896 | 0.0091 | 5.35E-09 |
| 89 | rs3791710 | C | T | -0.0600033 | 0.0108 | 3.02E-08 |
| 90 | rs3795310 | T | C | -0.0509972 | 0.0087 | 5.75E-09 |
| 91 | rs3802924 | C | A | -0.0736036 | 0.0108 | 9.58E-12 |
| 92 | rs3814883 | T | C | -0.0670977 | 0.0087 | 1.58E-14 |
| 93 | rs3824451 | C | T | 0.0655951 | 0.0118 | 2.54E-08 |
| 94 | rs4129585 | C | A | -0.0749962 | 0.0087 | 5.11E-18 |
| 95 | rs4575535 | G | A | 0.0557982 | 0.0096 | 5.77E-09 |
| 96 | rs4632195 | T | C | 0.0471964 | 0.0086 | 4.59E-08 |
| 97 | rs4636654 | A | G | -0.0483043 | 0.0089 | 4.89E-08 |
| 98 | rs4653164 | T | C | 0.0511038 | 0.0092 | 3.08E-08 |
| 99 | rs4700418 | G | C | 0.0701972 | 0.0087 | 5.37E-16 |
| 100 | rs4702 | A | G | -0.0843044 | 0.0089 | 2.79E-21 |
| 101 | rs4766428 | T | C | 0.0750038 | 0.0089 | 3.93E-17 |
| 102 | rs4779050 | G | T | -0.0579953 | 0.0089 | 7.27E-11 |
| 103 | rs4812325 | A | G | 0.0719042 | 0.0089 | 8.96E-16 |
| 104 | rs4921741 | G | A | 0.0559991 | 0.0098 | 1.21E-08 |
| 105 | rs498591 | T | A | 0.0724954 | 0.0121 | 2.11E-09 |
| 106 | rs500102 | C | T | -0.0517002 | 0.0088 | 4.87E-09 |
| 107 | rs505061 | A | C | 0.0534957 | 0.0086 | 5.80E-10 |
| 108 | rs56205728 | A | G | 0.0630037 | 0.0097 | 1.01E-10 |
| 109 | rs56335113 | G | A | -0.064701 | 0.0094 | 6.02E-12 |
| 110 | rs57433322 | G | C | -0.0830996 | 0.0139 | 1.99E-09 |
| 111 | rs5751191 | C | T | 0.0655951 | 0.0086 | 3.00E-14 |
| 112 | rs58120505 | C | T | -0.089603 | 0.0088 | 2.24E-24 |
| 113 | rs6001259 | T | C | 0.1915 | 0.0348 | 3.70E-08 |
| 114 | rs6010045 | C | T | 0.0548998 | 0.0095 | 7.44E-09 |
| 115 | rs60135207 | T | G | -0.0495994 | 0.0088 | 1.53E-08 |
| 116 | rs61937595 | T | C | -0.130098 | 0.0162 | 1.15E-15 |
| 117 | rs62018952 | C | T | 0.0584027 | 0.0097 | 1.94E-09 |
| 118 | rs62183855 | C | A | -0.0660967 | 0.0111 | 2.66E-09 |
| 119 | rs634940 | T | G | 0.0663962 | 0.0099 | 1.78E-11 |
| 120 | rs6482437 | C | A | 0.0989036 | 0.0142 | 3.33E-12 |
| 121 | rs6538539 | T | G | -0.0567961 | 0.0086 | 4.43E-11 |
| 122 | rs6546857 | G | A | 0.0603978 | 0.0102 | 2.74E-09 |
| 123 | rs6549963 | C | T | -0.0483043 | 0.0088 | 4.31E-08 |
| 124 | rs6673880 | G | A | 0.062301 | 0.0091 | 7.19E-12 |
| 125 | rs6715366 | A | G | 0.0540972 | 0.0097 | 2.49E-08 |
| 126 | rs6721531 | T | A | -0.0517002 | 0.0091 | 1.47E-08 |
| 127 | rs6798742 | G | A | 0.0610991 | 0.0093 | 4.57E-11 |
| 128 | rs6943762 | C | T | -0.105098 | 0.0132 | 1.57E-15 |
| 129 | rs6974218 | C | A | -0.0548953 | 0.0089 | 6.80E-10 |
| 130 | rs6984242 | A | G | -0.0546965 | 0.0087 | 3.85E-10 |
| 131 | rs708228 | T | C | 0.0527997 | 0.0091 | 6.56E-09 |
| 132 | rs7112616 | C | T | -0.0522034 | 0.0086 | 1.52E-09 |
| 133 | rs7113199 | C | A | -0.0522983 | 0.0094 | 2.80E-08 |
| 134 | rs7251 | G | C | -0.0641009 | 0.0094 | 8.29E-12 |
| 135 | rs72802868 | T | G | -0.0691995 | 0.0096 | 4.55E-13 |
| 136 | rs728055 | A | T | -0.0673969 | 0.009 | 8.85E-14 |
| 137 | rs72943392 | C | G | 0.0534957 | 0.0096 | 2.39E-08 |
| 138 | rs72986630 | T | C | 0.112296 | 0.0179 | 3.59E-10 |
| 139 | rs73229090 | A | C | -0.102602 | 0.0142 | 4.34E-13 |
| 140 | rs73292401 | A | T | 0.0676045 | 0.0109 | 5.48E-10 |
| 141 | rs7515363 | T | C | -0.0535029 | 0.0089 | 1.84E-09 |
| 142 | rs7575796 | G | A | -0.0963006 | 0.0172 | 2.07E-08 |
| 143 | rs7634476 | G | A | 0.0577033 | 0.0088 | 5.46E-11 |
| 144 | rs7647398 | T | C | -0.0774979 | 0.0109 | 1.07E-12 |
| 145 | rs778371 | G | A | 0.0806029 | 0.0095 | 1.49E-17 |
| 146 | rs7798283 | G | T | -0.074003 | 0.0134 | 3.49E-08 |
| 147 | rs79210963 | C | T | 0.0856015 | 0.0137 | 4.14E-10 |
| 148 | rs79445414 | C | T | 0.1234 | 0.0222 | 2.80E-08 |
| 149 | rs8055219 | A | G | 0.0665031 | 0.0101 | 5.69E-11 |
| 150 | rs9304548 | A | C | -0.0567016 | 0.01 | 1.59E-08 |
| 151 | rs9318627 | C | A | -0.0611986 | 0.0088 | 4.35E-12 |
| 152 | rs9454727 | G | A | -0.054403 | 0.0098 | 3.35E-08 |
| 153 | rs9461916 | C | T | 0.0532953 | 0.0088 | 1.64E-09 |
| 154 | rs9636107 | G | A | 0.0698969 | 0.0086 | 5.11E-16 |
| 155 | rs9687282 | G | T | 0.0525994 | 0.0091 | 7.33E-09 |
| 156 | rs9876421 | T | C | 0.0625033 | 0.0092 | 9.19E-12 |
